# Supplementary material for: Dissipation and resonance frequency shift of a resonator magnetically coupled to a semiclassical spin
Source: Sci Rep. 2017 Feb 10;7:42239. doi: 10.1038/srep42239 (PMC5301499; doi:10.1038/srep42239)
Supplement: Supplementary Information [file srep42239-s1.pdf]

APPENDICES FOLLOWING: DISSIPATION AND RESONANCE FREQUENCY SHIFT OF A RESONATOR MAGNETICALLY COUPLED TO A SEMICLASSICAL SPIN

J. M. de Voogd, J. J. T. Wagenaar and T. H. Oosterkamp

Appendix A: Resonator - Semiclassical Spin Lagrangian

The semiclassical magnetic moment $\boldsymbol{\mu}$ can be seen as a vector with an azimuth ϕ and a polar angle θ, where the poles of the spherical coordinate system ($\theta = 0°$ and $180°$) lie on the axis parallel to the magnetic field. θ and $\dot{\phi}$ can be seen as the two degrees of freedom that a spin has. Then the Lagrangian $L = \boldsymbol{\mu} \cdot \boldsymbol{B}(q) + S\hbar\dot{\phi}\cos\theta$ reveals the Bloch equations for a spin-S particle, but then without decay and for magnetic moment instead of magnetization. The last term of the Lagrangian describes the internal dynamics of the spin. Substituting this into the full Lagrangian in Eq. 1, we find

$$L = \frac{1}{2}m\dot{q}^2 - \frac{1}{2}kq^2 + \boldsymbol{\mu} \cdot \boldsymbol{B}(q) + S\hbar\dot{\phi}\cos\theta. \tag{A1}$$

Appendix B: Equilibrium magnetic moment

By definition of the equilibrium vector we can state that $-\boldsymbol{\mu}_\infty \cdot \boldsymbol{B} = \langle E \rangle$, where $\langle E \rangle$ is the equivalent ensemble average for the energy, or for a single spin the averaged energy over all the points in time with equal q. The limited energy levels make it easy to calculate the average energy: For spin-S there are $2S+1$ energy levels with energies $E_k = -kg_s\mu_s\,|\boldsymbol{B}|$ with $k = -S, -S+1, \ldots, S$. Using the relation between internal energy and the canonical partition function, this results in

$$\boldsymbol{\mu}_\infty = \mu_s\Big(\,(2S+1)\coth\big(\,(2S+1)\,\beta\mu_s\,|\boldsymbol{B}|\,\big) - \coth\big(\beta\mu_s\,|\boldsymbol{B}|\,\big)\Big)\hat{\boldsymbol{B}} \tag{B1}$$

$$\overset{S=\frac{1}{2}}{=} \mu_s\tanh\left(\beta\mu_s\,|\boldsymbol{B}|\right)\hat{\boldsymbol{B}}. \tag{B2}$$

This result is also known as the Brillouin function for the Zeeman energy. The imposed direction $\hat{\boldsymbol{B}}$ follows from Curie's law. The result might be different when the spin has (strong) interaction with its neighbors and when this leads to anisotropic effects, although some of these effects might be included in the q independent part of $\boldsymbol{B}$.

Appendix C: Zeroth order solution

If the magnetic field generated by the oscillating magnet is given by $\boldsymbol{B}(\boldsymbol{r})$ in the magnet's rest frame, then in the laboratory frame the magnetic field is $\boldsymbol{B}(\boldsymbol{r} - \lambda\boldsymbol{q})$. Around the spin position $\boldsymbol{r}_s$ the magnetic field is

$$\boldsymbol{B} = \boldsymbol{B}_0 - q\boldsymbol{B}' + \frac{1}{2}q^2\boldsymbol{B}'' + \ldots. \tag{C1}$$

Here $\boldsymbol{B}_0 \equiv \boldsymbol{B}(\boldsymbol{r}_s)$, $\boldsymbol{B}' \equiv \left.\frac{\partial \boldsymbol{B}}{\partial q}\right|_{\boldsymbol{r}=\boldsymbol{r}_s}$ and $\boldsymbol{B}'' \equiv \left.\frac{\partial^2 \boldsymbol{B}}{\partial^2 q}\right|_{\boldsymbol{r}=\boldsymbol{r}_s}$.
Next we substitute $q \to \lambda q$ and expand $\boldsymbol{\mu}_\infty$ for spin-$\frac{1}{2}$ up to first order in λ and omit higher order terms

$$\boldsymbol{\mu}_\infty = \mu_s\tanh\left(\beta\mu_s B_0\right)\hat{\boldsymbol{B}}_0 - q\left(\tanh\left(\beta\mu_s B_0\right)P_\perp + \frac{\beta\mu_s B_0}{\cosh^2\left(\beta\mu_s B_0\right)}P_\parallel\right)\frac{\boldsymbol{B}'}{B_0}, \tag{C2}$$

where $P_\parallel$ and $P_\perp$ are projections parallel and perpendicular to the $\boldsymbol{B}_0$ field respectively, i.e. $P_\parallel \equiv \hat{\boldsymbol{B}}_0\hat{\boldsymbol{B}}_0^T$ and $P_\perp \equiv \mathbb{1} - \hat{\boldsymbol{B}}_0\hat{\boldsymbol{B}}_0^T$. We also set $q \to \lambda q$ into Eqs. 3 and C1 and set $\lambda \to 0$ to get the differential equation to solve for $\boldsymbol{\mu}_0$:

$$\dot{\boldsymbol{\mu}}_0 = \left(\gamma_s B_{0\times} - \frac{1}{T_2}P_\perp - \frac{1}{T_2}P_\parallel\right)\boldsymbol{\mu}_0 + \frac{\mu_s}{T_1}\tanh\left(\beta\mu_s B_0\right)\hat{\boldsymbol{B}}_0, \tag{C3}$$

where the $\times$ subscript denotes an antisymmetric matrix such that $A_\times \boldsymbol{v} \equiv \boldsymbol{v} \times \boldsymbol{A}$ for any vector $\boldsymbol{v}$ and $\boldsymbol{A}$.

Let $\boldsymbol{M}(s) \equiv \int_0^\infty \mathrm{e}^{-st}\boldsymbol{\mu}(t)\,\mathrm{d}t$ be the Laplace transform of the magnetic moment and apply the necessary linear algebra to get

$$\boldsymbol{M}_0(s) = \left(\frac{\left(s+\frac{1}{T_2}\right)P_\perp + \omega_s \hat{B}_{0\times}}{\left(s+\frac{1}{T_2}\right)^2 + \omega_s^2} + \frac{P_\parallel}{s+\frac{1}{T_1}}\right)\left(\frac{1}{s}\frac{\mu_s}{T_1}\tanh\left(\beta\mu_s B_0\right)\hat{\boldsymbol{B}}_0 + \boldsymbol{\mu}(0)\right), \tag{C4}$$

with $\omega_s \equiv \gamma_s B_0$. The inverse Laplace transform yields the general solution for $\boldsymbol{\mu}_0$ in the time-domain:

$$\boldsymbol{\mu}_0(t) = \mu_s\left(1-\mathrm{e}^{-t/T_1}\right)\tanh\left(\beta\mu_s B_0\right)\hat{\boldsymbol{B}}_0 + \begin{pmatrix} \mathrm{e}^{-t/T_2}\cos(\omega_s t) & -\mathrm{e}^{-t/T_2}\sin(\omega_s t) & 0 \\ \mathrm{e}^{-t/T_2}\sin(\omega_s t) & \mathrm{e}^{-t/T_2}\cos(\omega_s t) & 0 \\ 0 & 0 & \mathrm{e}^{-t/T_1} \end{pmatrix}\boldsymbol{\mu}(0). \tag{C5}$$

To retrieve some intuition for the results we choose to present the last term as a matrix which is given in a non-rotating Cartesian basis with $\hat{\boldsymbol{z}} = \hat{\boldsymbol{B}}_0$.
To estimate the static displacement we use $\boldsymbol{\mu}_0(\infty)$, which is of course the same as $\boldsymbol{\mu}_\infty(q=0)$, to find the change of equilibrium position

$$q \to q - \frac{\boldsymbol{\mu}_0\cdot\boldsymbol{B}'}{k+\delta k} \approx q - \frac{\mu_s}{k}\tanh\left(\beta\mu_s B_0\right)\hat{\boldsymbol{B}}_0\cdot\boldsymbol{B}', \tag{C6}$$

where in the last step we neglected δk, the effective extra stiffness coming from the terms linear in q.

Appendix D: First order solution

As argued in the main text, we can ignore the terms that decay or depend on initial conditions. As a consequence we can take $\boldsymbol{\mu}_0 = \mu_s\tanh\left(\beta\mu_s B_0\right)\hat{\boldsymbol{B}}_0$. This leads immediately to one of the interaction terms. Taking $\mathcal{F}\left\{-q\boldsymbol{\mu}_0\cdot\boldsymbol{B}''\right\} = \kappa_1\tilde{q}(\omega)$ with $\tilde{q}(\omega) = \mathcal{F}\{q(t)\}$ we arrive at

$$\kappa_1 = -\mu_s\left|\boldsymbol{B}''_{\parallel\hat{\boldsymbol{B}}_0}\right|\tanh\left(\beta\mu_s B_0\right), \tag{D1}$$

where $\left|\boldsymbol{B}''_{\parallel\hat{\boldsymbol{B}}_0}\right| = \boldsymbol{B}''\cdot\hat{\boldsymbol{B}}_0$.
Next, we need to find $\boldsymbol{\mu}_1$. Again this is done by substituting $q \to \lambda q$ and extracting the terms that are linear in λ only. We find

$$\begin{aligned} \dot{\boldsymbol{\mu}}_1 = &\left(\gamma_s B_{0\times} - \frac{1}{T_2}P_\perp - \frac{1}{T_2}P_\parallel\right)\boldsymbol{\mu}_1 \\ &+ q(t)\left(\left(\frac{1}{T_2}-\frac{1}{T_1}\right)C - \gamma_s B'_\times\right)\boldsymbol{\mu}_0 \\ &- q(t)\frac{\mu_s}{B_0 T_1}\left(\tanh\left(\beta\mu_s B_0\right)P_\perp + \frac{\beta\mu_s B_0}{\cosh^2\left(\beta\mu_s B_0\right)}\right)\hat{\boldsymbol{B}}', \end{aligned} \tag{D2}$$

where $C \equiv \frac{1}{B_0}\left(\hat{\boldsymbol{B}}_0\boldsymbol{B}'^T P_\perp + P_\perp\boldsymbol{B}'\hat{\boldsymbol{B}}_0^T\right)$.
The first line is the same as in Eq. C3 and therefore leads to the same matrix as in Eq. C4 using the same non-rotating Cartesian basis with $\boldsymbol{z} = \hat{\boldsymbol{B}}_0$. This leads to

$$\begin{aligned} \boldsymbol{M}_1(s) = &\left(\frac{\left(s+\frac{1}{T_2}\right)P_\perp + \omega_s\hat{B}_{0\times}}{\left(s+\frac{1}{T_2}\right)^2+\omega_s^2} + \frac{P_\parallel}{s+\frac{1}{T_1}}\right)\cdot \\ &\left(\tanh\left(\beta\mu_s B_0\right)\left(\left(\frac{1}{T_2}-\frac{2}{T_1}\right)P_\perp\boldsymbol{B}' - \omega_s\hat{\boldsymbol{B}}_0\times\boldsymbol{B}'\right) - \frac{\beta\mu_s B_0}{\cosh^2\left(\beta\mu_s B_0\right)}P_\parallel\boldsymbol{B}'\right)\frac{\mu_s}{B_0 T_1}Q(s), \end{aligned} \tag{D3}$$

with $\boldsymbol{M}_1(s)$ and $Q(s)$ being the Laplace transform of $\boldsymbol{\mu}_1(t)$ and $q(t)$ respectively.
$\boldsymbol{M}_1$, and thus $\boldsymbol{\mu}_1$, can be easily split in a part that is parallel and perpendicular to $\hat{\boldsymbol{B}}_0$. It follows from Eq. 8 that we need specifically the product $\boldsymbol{\mu}_1 \cdot \boldsymbol{B}'$ for the interaction term. So let us write $\mathcal{F}\left\{\boldsymbol{\mu}_1 \cdot \boldsymbol{B}'\right\} = \tilde{q}(\omega)\left(\kappa_2 + \kappa_3\right)$ where κ_2 and κ_3 come from the parallel and perpendicular parts of $\boldsymbol{\mu}_1$ respectively. Finally we move to the Fourier domain, which is possible since all poles lie in the $\mathrm{Re}(s) < 0$ regime. This leads to

$$\kappa_2 = -\frac{\mu_s}{B_0}\left|\boldsymbol{B}'_{\parallel \hat{\boldsymbol{B}}_0}\right|^2 \frac{\beta\mu_s B_0}{\cosh^2\left(\beta\mu_s B_0\right)}\frac{1}{1+i\omega T_1}, \tag{D4}$$

where $\left|\boldsymbol{B}'_{\parallel \hat{\boldsymbol{B}}_0}\right|^2 = \boldsymbol{B}'^T P_{\parallel}\boldsymbol{B}'$.
For κ_3 we find

$$\kappa_3 = -\frac{\mu_s}{B_0}\left|\boldsymbol{B}'_{\perp \hat{\boldsymbol{B}}_0}\right|^2 \tanh\left(\beta\mu_s B_0\right)\left(1 - \frac{2\frac{T_2}{T_1} - \left(\omega T_2\right)^2 + i\omega T_2\left(1 + 2\frac{T_2}{T_1}\right)}{\left(1 + i\omega T_2\right)^2 + \left(\omega_s T_2\right)^2}\right), \tag{D5}$$

where $\left|\boldsymbol{B}'_{\perp \hat{\boldsymbol{B}}_0}\right|^2 = \boldsymbol{B}'^T P_{\perp}\boldsymbol{B}'$.

Appendix E: Spin bath integral

We want to integrate the κ-terms over a volume of spins that has a constant spin density ρ. Before we start, we split the volume in a component perpendicular ($\mathcal{V}_{\boldsymbol{q}\perp}$) and along ($\mathcal{V}_{q_\parallel}$) the resonator movement, such that we can write the integration as

$$\kappa = \rho\int_{\mathcal{V}_{q_\perp}} \mathrm{d}\boldsymbol{q}_\perp \int_{\mathcal{V}_{q_\parallel}} \mathrm{d}q\left(\kappa_1 + \kappa_2 + \kappa_3\right). \tag{E1}$$

Using the identities

$$\frac{\partial}{\partial q}\tanh\left(\beta\mu_s B\right)\bigg|_{\boldsymbol{r}=\boldsymbol{r}_s} = \frac{\beta\mu_s}{\cosh^2\left(\beta\mu_s B_0\right)}\left|\boldsymbol{B}'_{\parallel \hat{\boldsymbol{B}}_0}\right|, \qquad \text{and} \tag{E2}$$

$$\frac{\partial}{\partial q}\left(\hat{\boldsymbol{B}}\cdot\frac{\partial}{\partial q}\boldsymbol{B}\right)\bigg|_{\boldsymbol{r}=\boldsymbol{r}_s} = \left|\boldsymbol{B}''_{\parallel \hat{\boldsymbol{B}}_0}\right| + \frac{1}{B_0}\left|\boldsymbol{B}'_{\perp \hat{\boldsymbol{B}}_0}\right|^2, \tag{E3}$$

we find by partial integration

$$\int_{\mathcal{V}_{q_\parallel}} \mathrm{d}q\ \kappa_2(T_1 = 0) = -\mu_s\tanh\left(\beta\mu_s B\right)\left|\boldsymbol{B}'_{\parallel \hat{\boldsymbol{B}}_0}\right|\bigg|_{\partial\mathcal{V}_{q_\parallel}} - \int_{\mathcal{V}_{q_\parallel}} \mathrm{d}q\ \left(\kappa_1 + \kappa_3(T_2 = 0)\right). \tag{E4}$$

Using this result and the expressions for κ_2 and κ_3 (Eq. D4 and D5), we can write the total integral

$$\begin{aligned}\kappa =& \rho\beta\mu_s^2\frac{\left(\omega T_1\right)^2 + i\omega T_1}{1 + (\omega T_1)^2}\int_{\mathcal{V}_{q_\perp}} \mathrm{d}\boldsymbol{q}_\perp \int_{\mathcal{V}_{q_\parallel}} \mathrm{d}q\frac{\left|\boldsymbol{B}'_{\parallel \hat{\boldsymbol{B}}_0}\right|^2}{\cosh^2\left(\beta\mu_s B_0\right)} \\ &+ \rho\mu_s\frac{2\frac{T_2}{T_1} - \left(\omega T_2\right)^2 + i\omega T_2\left(1 + 2\frac{T_2}{T_1}\right)}{\left(1 + i\omega T_2\right)^2 + \left(\omega_s T_2\right)^2}\int_{\mathcal{V}_{q_\perp}} \mathrm{d}\boldsymbol{q}_\perp \int_{\mathcal{V}_{q_\parallel}} \mathrm{d}q\tanh\left(\beta\mu_s B_0\right)\frac{1}{B_0}\left|\boldsymbol{B}'_{\perp \hat{\boldsymbol{B}}_0}\right|^2 \\ &- \rho\int_{\mathcal{V}_{q_\perp}} \mathrm{d}\boldsymbol{q}_\perp\ \mu_s\tanh\left(\beta\mu_s B\right)\left|\boldsymbol{B}'_{\parallel \hat{\boldsymbol{B}}_0}\right|\bigg|_{\partial\mathcal{V}_{q_\parallel}}.\end{aligned} \tag{E5}$$
